# Breaking New Ground in Palliative Care: Examining the Impact of Al Ain – Palliative Care Outreach Program on Patients With Advanced Cancer in the United Arab Emirates

**DOI:** 10.7759/cureus.36756

**Published:** 2023-03-27

**Authors:** Mehad Araki, Nandan M Shanbhag, Khaled H Al Qawasmeh, Mohammad M Saleh, Muhammad Z Javed, Afroz Samad

**Affiliations:** 1 Nursing, Tawam Hospital, Al Ain, ARE; 2 Radiation Oncology/Palliative Care, Tawam Hospital, Al Ain, ARE; 3 Oncology, Tawam Hospital, Al Ain, ARE; 4 Oncology/Palliative Care, Tawam Hospital, Al Ain, ARE

**Keywords:** spiritual well-being, psychological well-being, physical well-being, palliative care instrument, consumer quality (cq) index, united arab emirates, advanced cancer, quality of care, palliative outreach program, palliative care

## Abstract

Introduction

This study aimed to evaluate the effectiveness of the Palliative Outreach Program in improving the quality of palliative care for patients with advanced cancer in a Tertiary Hospital in the Al Ain region of the United Arab Emirates (UAE).

Methods & Material

One hundred patients who met the inclusion criteria were included in the study and administered the patient version of the Consumer Quality (CQ) Index Palliative Care Instrument to assess their perception of the quality of care they received. The demographics, diagnosis, and questionnaire responses were analyzed to determine the effectiveness of the Palliative Outreach Program.

Results

A total of one hundred patients met the criteria for the study. Most patients were above 50, female, female, Non-Emiratis, and had high school certificates. The top three cancer diagnoses were breast (22%), lung (15%), and head & neck (13%). The patients reported high levels of support from their caregivers regarding physical, psychological, and spiritual well-being, as well as information and expertise. The mean scores for most variables were favorable, except for information (mean = 2.9540, SD= 0.25082) and general appreciation (mean = 6.7150, sd = 0.82344). Overall, the patients rated the care they received positively, with high mean scores for physical/psychological well-being (mean = 3.4950, SD = 0.28668), autonomy (mean = 3.7667, SD= 0.28623), privacy (mean = 3.6490, SD = 0.23159), and spiritual well-being (mean =3.7500, SD = 0.54356). The patients would recommend their caregivers to others in similar situations.

Discussion

The findings demonstrate that the Palliative Outreach Program effectively improves the quality of palliative care for patients with advanced cancer in the UAE. The CQ Index Palliative Care Instrument proved a novel method for assessing palliative care quality from patients' perspectives. However, there is room for improvement in providing more favorable information and general appreciation outcomes. Caregivers should focus on all areas to enhance their physical/psychological well-being, autonomy, privacy, spiritual well-being, expertise, and general appreciation of their patients.

Conclusion

In conclusion, the Palliative Outreach Program is an effective intervention to improve the quality of palliative care for patients with advanced cancer in the UAE. The patients reported high levels of support from their caregivers in all aspects of care, except for information and general appreciation. These findings provide valuable insights into the effectiveness of palliative care interventions and highlight the need for continued efforts to improve the quality of care for patients with advanced cancer.

## Introduction

The World Health Organisation defines palliative care as "an approach that improves the quality of life of patients and their families facing the problems associated with life-threatening illness through the prevention and relief of suffering using early identification and impeccable assessment and treatment of pain and other problems, physical, psychosocial, and spiritual" and it places a premium on delivering patient-centered care [1]. A fundamental component of this ethos is a comprehensive understanding of the quality of care from the patient's perspective. However, traditional approaches to measuring the quality of care, such as satisfaction surveys, are plagued by several drawbacks, including the need for more specificity in defining satisfaction, an inherent dependence on the individual's expectations, and the possibility of eliciting socially desirable responses. In response to these issues, a new generation of quality instruments has emerged based on measuring actual care experiences. The Consumer Quality Index (CQ-index) represents one such structured questionnaire, which assesses the quality of care from the viewpoint of care users [2].

Distinct from conventional satisfaction-based measurements, the CQ-indices approach centers around evaluating a patient's care experiences in tandem with their expectations [3]. By juxtaposing the patient's experiences against their expectations, the CQ-index facilitates a more accurate reflection of the quality of care and provides a clear roadmap for quality improvement interventions. The CQ indices have been developed and used for diverse target groups, including patients with breast cancer, rheumatoid arthritis, and those receiving long-term care [4].

The need for an excellent and comprehensive tool for assessing the quality of palliative care resulted in the creation of the CQ-index Palliative Care (CQ-index PC) by the Dutch Ministry of Health in 2007 [5]. The CQ-index PC distinguishes itself from extant instruments by avoiding traditional satisfaction-based metrics and focusing on the importance of the patient's actual experiences. The CQ-index PC combines questions on actual experiences with questions about how vital care aspects are to the patient, providing a more holistic and nuanced approach to quality assessment. By scrutinizing the relationship between 'experience scores' and 'importance scores,' the CQ-index PC offers granular insights into which care areas require the most urgent attention to effect quality improvement.

Palliative care outreach services aim to improve patient's quality of life and end-of-life care in their preferred location, reducing the burden on family caregivers and improving communication between healthcare providers [6]. Several studies have explored the impact of these services on improving the quality of care for patients with advanced illnesses. One study showed that outreach services reduced healthcare costs, improved patient and family satisfaction, and decreased readmission rates in ICU [7]. Another study found that palliative care interventions for people with advanced dementia can address physical, psychological, and spiritual needs, leading to improved quality of life for both patients and caregivers [8]. A palliative care outreach team in India also demonstrated improved symptom management, emotional support, and communication with patients and families [9]. A systematic review and meta-analysis of palliative care outreach services for patients with cancer found improved symptom management, quality of life, and patient and family satisfaction [10]. 

Tawam Hospital stands out as a beacon of hope for those needing oncology services in the United Arab Emirates (UAE). The structured tertiary hospital serves as a national and regional referral center for oncology services. It has taken significant strides to provide patients with the care and support they need during their journey. Tawam Hospital has the first Palliative Care Unit in the country, with a bed capacity of only 11 beds and the ability to serve up to 20 patients; therefore, the Palliative Care Outreach Program and Consultation Service were initiated to ensure that oncology patients receive the best possible care and palliative care service is extended to serve a more significant number of population within the hospital.

 This paper presents the latest innovative approach to measuring patient experiences in the palliative care outreach program, using the CQ-index PC as the primary instrument. This paper provides insights into patients' care experiences, quality aspects that patients value, and the care aspects requiring the most critical attention to enhance the quality of care. The CQ-index PC is a practical, patient-centric instrument for measuring quality indicators from the patient's perspective. Its adoption can offer vital quality information for healthcare organizations, patients, relatives, and external entities such as the Health Care Inspectorate. The CQ-index PC is a powerful tool for assessing the quality of palliative care and facilitating effective quality improvement interventions in diverse care settings. 

## Materials and methods

The study presented has outlined clear inclusion and exclusion criteria for participants. To be eligible for inclusion, patients must receive Palliative Care Service, have a life expectancy of six months or less, be 18 years or older, and consent to participate. Furthermore, patients must be physically and mentally competent and located outside the palliative care unit, with a study duration of one week. Exclusion criteria include refusal to participate, patients in the terminal phase, and those who are mentally or cognitively incompetent. Non-probability sampling, specifically convenience sampling, was used to obtain a sample size of 100 patients located anywhere in Tawam Hospital except the palliative care unit. The research design is a quantitative non-experimental cross-sectional design with descriptive statistics, which would provide valuable insights into the patient population receiving palliative care services in Tawam Hospital.

The study uses the comprehensive Consumer Quality Index Palliative Care (CQ-index PC) questionnaire. The CQ-index PC is a comprehensive questionnaire consisting of eight components: physical well-being, Psychological well-being, Autonomy, Privacy, Spiritual well-being, Information, Expertise of the caregiver, and General Appreciation. The questionnaire comprises 34 questions, with essential items and experiences items measuring patient satisfaction with care. Patients rate the importance of care items on a four-point Likert scale and their actual care experiences on a four-point Likert scale, ranging from 'no/never' to 'always'. If a patient has not experienced a specific symptom, they can choose the 'not applicable.'

The Tawam Human Research Ethics Committee approved the study, and the IRB approval number is THREC-668. The data were analyzed using Statistical Package for Social Science (SPSS), version 26, with descriptive statistics computed for the background characteristics, including frequencies, percentages, means (with 95% confidence intervals), and demographic data. 

## Results

The study reports the patients' experiences with palliative care using descriptive statistics such as percentages, means, and standard deviations. Percentages are used to report demographic characteristics, while means and standard deviations summarize patient perceptions of different aspects of care. The study provides a detailed picture of the patient's experiences, including areas where care was perceived positively, and improvements could be made. Using descriptive statistics, the study provides a concise and clear summary of the patient's experiences with palliative care.

Demographics

Age

**Figure 1:**
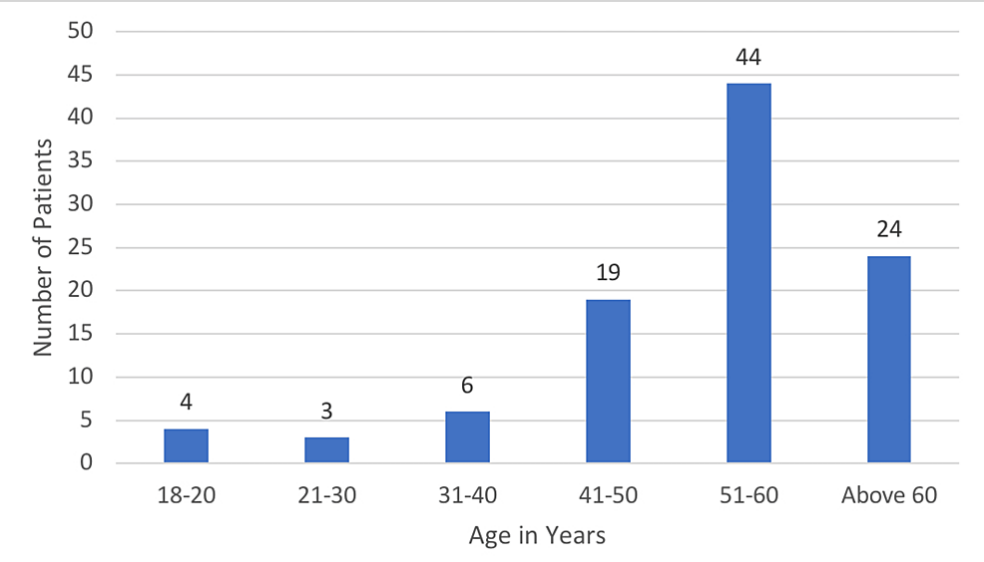
Age Distribution

Most patients (44%) were aged between 51-60 years, followed by those above 60 years with 24% (Figure 1).

Gender, Religion, Marital Status, and Nationality

There were 57 females and 43 males, with the majority (68%) of the respondents being non-Emiratis, and there were only 32% Emiratis. This denotes the wide nationalities in the United Arab Emirates, which caters to a wide variety of people worldwide. Most (69%) of the patients were Islamic, and close to 67% were married (Table 1).

**Table 1:** Gender, Religion, Marital Status, and respondents nationality.

Gender		n	%
	Male	43	43
	Female	57	57
	Total	100	100
Religion			
	Muslims	69	69
	Christians	18	18
	Others	13	13
	Total	100	100
Marital Status			
	Married	67	67
	Single	10	10
	Widower	17	17
	Divorcee	6	6
	Total	100	100
Nationality			
	Emirati	68	68
	Non-Emirati	32	32
	Total	100	100

Primary Malignancy

**Figure 2:**
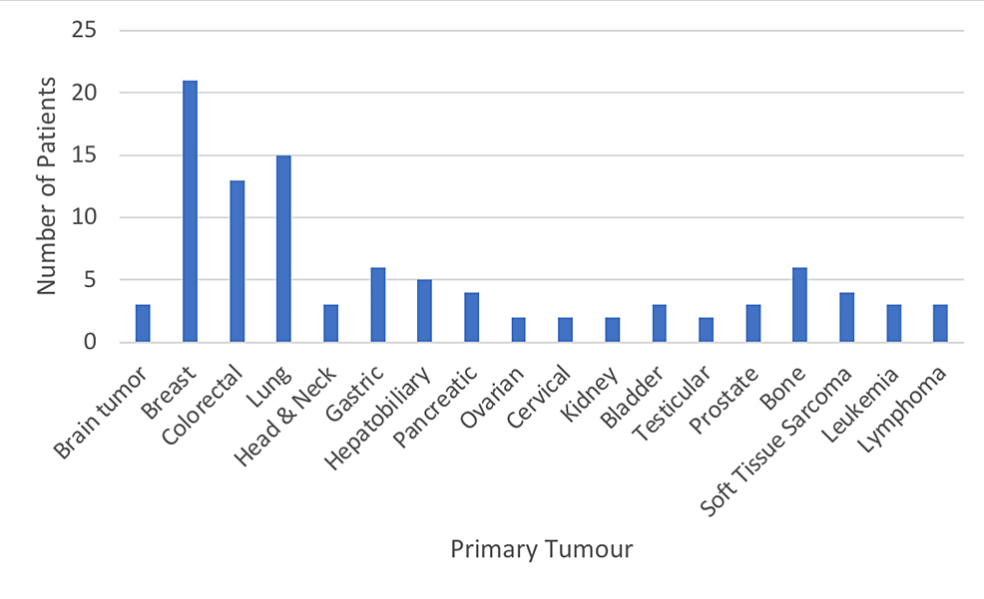
Primary Diagnosis

Breast Cancer (22%) was the most common primary diagnosis, followed by lung (15%) and colorectal (13%) (Figure 2).

Literacy and Education

**Figure 3:**
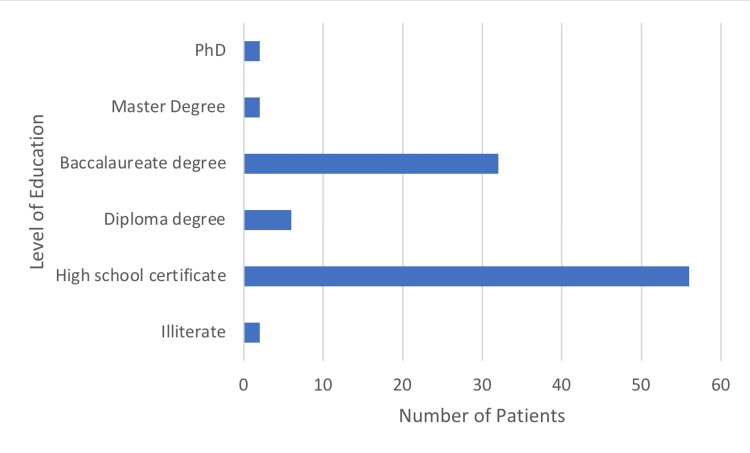
Education and Literacy

Almost all patients were literate, and most had a high school certificate and above (Figure 3).

Physical Well Being 

When asked about Physical Well Being, the results suggest that patients receive significant support from their caregivers, as evidenced by the favorable responses to all questions, with over 50% of participants indicating agreement or satisfaction (Table 2).

**Table 2:** Questions to patients about the support they get from their HCP when experiencing specific symptoms. HCP: Health Care Provider

		Never (%)	Sometimes (%)	Usually (%)	Always (%)	Not Applicable (%)	Total (%)
1	Do you receive support when you are in pain?	0	4	11	79	6	100
2	Do you receive support when feeling tired?	3	24	22	51	0	100
3	Do you receive support when you have shortness of breath?	1	3	9	69	18	100
4	Do you receive support when you are constipated?	0	2	14	77	7	100
5	Do your caregivers help you with your physical self-care?	0	0	9	75	16	100

Psychological Well Being

The psychological component of the questionnaire comprised two distinct sections, one on the timeliness of the healthcare provider's (HCP) response to patient needs and the other focused on the quality of the response provided. Across all measures related to psychological well-being, results indicated that over 50% of patients responded affirmatively, reporting good psychological well-being, which was attributed to the supportive care provided by their caregivers (Table 3, 4).

**Table 3:** Patient perception of Support and Communication with Caregivers in Palliative Care: Frequency of Support Received for Anxiety and Depression, and Communication about Emotional Well-being.

		Never (%)	Sometimes (%)	Usually (%)	Always (%)	Not Applicable (%)	Total (%)
7	Do you receive support when you feel anxious?	5	6	28	61	0	100
8	Do you receive support when you feel depressed?	3	29	27	39	2	100
15	Do you have the opportunity to talk to your caregivers about how you are feeling?	1	10	13	76	0	100

**Table 4:** Patient Perception of Caregiver Attitudes and Behaviors in Palliative Care: Frequency of Caregiver Politeness, Listening, Time, Seriousness, Interest, Warmth, and Attention to Relatives

		None of them (%)	Some of them (%)	Most of them (%)	All of them (%)	Total (%)
9	Are your caregivers polite to you?	0	1	8	91	100
10	Do your caregivers listen carefully to you?	0	6	5	89	100
11	Do your caregivers have enough time for you?	0	7	37	56	100
12	Do your caregivers take you seriously?	0	1	9	90	100
13	Do your caregivers show interest in your personal situation?	0	6	13	81	100
14	Do your caregivers have a ‘warm’ attitude?	0	2	17	81	100
16	Do your caregivers pay attention to your relative(s)?	1	7	16	76	100

Patient Autonomy & Privacy

Regarding patients' autonomy, results indicated that all participants acknowledged varying degrees of respect for their autonomy, with over 75% indicating that their autonomy was always respected. Furthermore, more than 90% of participants reported being consistently included and involved in decisions about their care (Table 5, 6). For privacy, two questions were asked; 56% of patients indicated that they always had the opportunity to be alone if they wanted to, while 61% indicated that it was always possible to talk to someone without being disturbed (Table 7).

**Table 5:** Autonomy and Inclusive Decision making

		Never (%)	Sometimes(%)	Usually (%)	Always (%)	N/A (%)	Total (%)
17	Do your caregivers give you a chance to plan your day?	0	4	17	76	3	100
18	Are you involved in decisions about your care?	0	2	7	91	0	100

**Table 6:** Autonomy and Personal Wishes

		None of them (%)	Some of them (%)	Most of them (%)	All of them (%)	Total (%)
19	Do your caregivers take your personal wishes into account?		8	21	71	100

**Table 7:** Privacy

		Never (%)	Sometimes (%)	Usually (%)	Always (%)	Not Applicable (%)	Total (%)
20	Do you get the opportunity to be alone if you want to be?	2	9	21	56	12	100
21	Is it possible to talk to someone without being disturbed if you want to?	2	12	22	61	3	100

Spiritual Well Being

On patient perceptions of spiritual care in palliative care, finding that caregiver respect for patient life stance was generally high (71% reported always being respected), as was access to spiritual counseling (35% reported always having access) (Table 8).

**Table 8:** Patient Perception of Spiritual Care in Palliative Care: Frequency of Caregiver Respect for Life Stance and Access to Spiritual Counseling.

		None of them (%)	Some of them (%)	Most of them (%)	All of them (%)	Not Applicable (%)	Total (%)
22	Do your caregivers respect your life stance?		1	10	71	18	100
		Never (%)	Sometimes (%)	Usually (%)	Always (%)	Not Applicable (%)	Total (%)
23	Do you have access to a counselor for spiritual problems? (e.g.aminister/priest or humanist counselor)	2	9	43	35	11	100

Healthcare Giver Communication

On patient perceptions of the communication and information received in palliative care, the results indicated that a majority of patients reported that their caregivers effectively communicated with them, explaining things in a way they could understand (91%) and avoiding contradictory information (93%) (Table 9). Additionally, patients reported receiving adequate information about their illness, treatment options, and the contact person for their care. Notably, over half of the patients reported receiving information about the advantages and disadvantages of various types of treatment (61%) (Table 10).

**Table 9:** Caregiver Communication

		Never (%)	Sometimes (%)	Usually (%)	Always (%)	Total (%)
24	Do your caregivers explain things to you in a way you can understand?	0	9	53	38	100
25	Do your caregivers give you contradictory information?	93	6	0	1	100

**Table 10:** Information Dissemination

		No, not at all (%)	A little (%)	Quite a lot (%)	Yes, entirely (%)	Total (%)
26	Do you receive information about the expected course of the illness?	3	8	59	30	100
27	Do you know who the contact person is for the care?	2	3	21	74	100
28	Do you receive information about the advantages and disadvantages of various types of treatment?	0	5	34	61	100

Healthcare giver competence

Patient Perspective

Close to 98% of the patients believed their healthcare provider had the necessary expertise and competence to care for them (Table 11). Results showed that most patients reported good coordination of care between different caregivers (77%) and received timely help when in need of care (59%). Moreover, most patients reported receiving help in good times in acute situations (73%) (Table 12).

**Table 11:** Healthcare Giver Expertise

		None of them (%)	Some of them (%)	Most of them (%)	All of them (%)	Total (%)
29	Do your caregivers have the necessary expertise?	0	2	15	83	100

**Table 12:** Patient Perception of Care Coordination and Timeliness in Palliative Care

		Never (%)	Sometimes (%)	Usually (%)	Always (%)	Not Applicable	Total (%)
30	Is there a good match between the cares provided by the different caregivers involved in looking after you?	0	6	17	77	0	100
31	Do you receive help in good time when you are in need of care?	2	19	59	20	0	100
32	Are you offered help in good time in acute situations?	0	3	11	73	13	100

General Appreciation for Care

When asked if the patients would recommend their caregivers to patients in a similar situation, close to 85% were satisfied with the care provided and would recommend the healthcare giver to other patients.

Descriptive Analysis

Finally, a descriptive analysis of the results showed that patients rated their physical well-being highest (mean = 3.4950, SD = 0.28668), followed by autonomy (mean = 3.7667, SD = 0.28623), and psychosocial well-being (mean = 3.6490, SD = 0.23159). Patients rated the information domain lowest (mean = 2.9540, SD = 0.25082) (Table 13).

**Table 13:** Descriptive Statistics

	N	Minimum	Maximum	Mean	Std. Deviation
Physical Well Being	100	2.50	4.17	3.4950	.28668
Psychosocial Well Being	100	3.00	4.00	3.6490	.23159
Autonomy	100	2.67	4.33	3.7667	.28623
Privacy	100	2.00	4.50	3.5900	.57022
Spiritual Well Being	100	1.50	5.00	3.7500	.54356
Information	100	2.20	3.60	2.9540	.25082
Expertise	100	2.75	4.25	3.6125	.30438
General Appreciation	100	3.50	7.50	6.7150	.82344

## Discussion

Palliative care is an approach to care that focuses on improving the quality of life of patients with life-limiting illnesses and their families by addressing their physical, psychological, social, and spiritual needs [11]. The goal of palliative care is to relieve suffering and improve the patient's quality of life rather than to cure the underlying illness. Palliative care is usually provided by a team of healthcare professionals, including physicians, nurses, social workers, and chaplains [12].

One important aspect of palliative care is communication with patients and their families, which is essential for ensuring that their needs and preferences are met, and for promoting patient-centered care [13]. Effective communication involves active listening, empathy, and the ability to provide information clearly and understandably. It also includes respecting patients' autonomy and involving them in decision-making about their care [14]. The results of this study indicate that patients received high-quality care from their caregivers in the palliative care outreach program, with high levels of satisfaction and positive perceptions of their care. Patients reported that their autonomy was always respected, and they were consistently involved in decisions about their care. Patients also reported high levels of satisfaction with the communication and information provided by their caregivers. These findings are consistent with previous research showing the importance of effective communication in palliative care [15].

Another essential aspect of palliative care is the management of physical symptoms, such as pain, nausea, and fatigue, among others. Effective symptom management is essential for improving patients' quality of life and reducing their suffering. One of the key findings of this study was that patients rated their physical well-being the highest, which suggests that their caregivers managed their physical symptoms well. Effective symptom management is a critical component of palliative care, as it can significantly improve patient's quality of life and reduce their suffering [16]. Various pharmacological and non-pharmacological interventions are available for symptom management, including pain management, nausea, and vomiting control, dyspnea management, and others [17]. The goal of symptom management in palliative care is not to cure the underlying illness but to improve the patient's quality of life and relieve their suffering.

Managing psychological and emotional distress in patients and their families is challenging and as important as managing physical symptoms. Patients with life-limiting illnesses often experience anxiety, depression, fear, and other psychological and emotional symptoms, significantly affecting their quality of life [18]. This study found that patients reported good psychological well-being attributed to the supportive care provided by their caregivers. This finding is consistent with previous research showing the importance of addressing patients' psychological and emotional needs in palliative care [19].

Spiritual care is another essential aspect of palliative care, which involves addressing patients' spiritual and existential concerns, such as their beliefs, values, and sense of meaning and purpose [20]. Spiritual care is particularly important for patients with life-limiting illnesses, as they often face existential questions and concerns about the meaning and purpose of their lives [21,22]. In this study, patients rated their spiritual well-being positively and felt their caregivers respected their life stance. However, access to spiritual counseling was reported to be relatively low, indicating the need for improvement in this area. 

Patient autonomy is another crucial aspect of palliative care, as it enables patients to make informed decisions about their care and treatment options and to maintain control over their lives [23]. In this study, the patients reported that their autonomy was always respected, and they were consistently involved in decisions about their care. This finding is consistent with the principles of patient-centered care, which emphasizes respecting patients' autonomy and involving them in decision-making about their care [24].

Patient and family education is another essential aspect of palliative care. It enables patients and their families to make informed decisions about their care and manage their symptoms and other concerns effectively. Patient and family education can include information about the patient's illness, treatment options, symptom management, and advanced care planning [25]. In this study, patients reported high levels of satisfaction with the communication and information provided by their caregivers, indicating the importance of effective patient and family education in palliative care.

Cultural and linguistic competence is another essential aspect of palliative care, particularly in diverse and multicultural societies. The study you provided found that most patients were non-Emiratis, indicating the need for culturally and linguistically competent care. Cultural and linguistic competence involves understanding and respecting patients' and their families' cultural beliefs, values, and practices and providing care sensitive to their cultural and linguistic needs [26]. Failure to provide culturally and linguistically competent care can lead to poor patient outcomes, including decreased patient and family satisfaction and poor communication and collaboration between healthcare providers and patients and their families [27].

Finally, healthcare provider competence and expertise are essential to palliative care, as they ensure that patients receive high-quality and safe care. Your study found that almost all patients believed their healthcare provider had the necessary expertise and competence to care for them, indicating the importance of healthcare provider competence in palliative care. Healthcare provider competence involves having the necessary knowledge, skills, and training to provide high-quality and safe care and the ability to communicate effectively with patients and their families [28].

Our study suggests that while most patients with advanced cancer receive palliative care that matches what they consider necessary, there is potential for improving care, particularly in addressing symptoms such as fatigue. We recommend giving more attention to possible symptoms and acknowledging that complete symptom control may not always be achievable, particularly in the final phase of the disease. The study also highlights the importance of tailoring information about the expected course of the illness to individual preferences, as preferences may differ between patients and over time.

However, the study acknowledges that providing information about the expected course of the illness may be difficult, as the course may only sometimes be predictable. The information provided should be broader than just information about the expected course of the disease. For instance, advance care planning should include opportunities for good care if a patient suffers from specific symptoms and discussing the patient's perspective on end-of-life decisions about (potentially) life-prolonging or life-shortening treatments. The study also highlights the usefulness of the CQ Index Palliative Care Instrument - Patient Version as a novel method for assessing palliative care quality from patients' perspectives. Organizations can use the findings to assess which care aspects have the highest priority for quality improvement within their organization.

General Practitioners (GP) and nurses are important in providing palliative care, and we recommend they provide emotional support and practical advice even when symptoms are difficult to control. We also suggest that GPs discuss patients' need for information about the expected course of their illness. Moreover, given the important role of GPs and home care nurses in palliative care, the study's results suggest that it could be helpful to have insight into the extent to which their perspective on respecting autonomy and information provision matches that of their patients with advanced cancer.

Limitations

We acknowledge some potential limitations to the study, and future studies may consider the following to overcome these limitations. The study may need more generalizability due to the relatively small sample size. With only 100 participants, it may be difficult to draw firm conclusions about the experiences of all palliative care patients. The study was conducted at a single center, which may limit the generalizability of the findings to other palliative care settings. Patients at other centers may have different experiences and perceptions of care.

The study had a predominantly Muslim and married population, which may not reflect the diversity of patients in other palliative care settings. Patients from other religious or cultural backgrounds, or those who are not married, may have different experiences and perceptions of care. The questionnaire used in the study focused on a limited set of domains, which may not fully capture the complexity of patients' experiences in palliative care. Additional domains such as financial burden, caregiver burden, and social support could have been included to provide a more comprehensive picture of patients' experiences.

The study had a cross-sectional design, which limits the ability to establish causality or determine changes over time. In the future, we are considering a longitudinal study design better suited to examine changes in patients' experiences and perceptions of care over time.

## Conclusions

In conclusion, palliative care is a multidisciplinary approach to care that aims to improve the quality of life of patients with life-limiting illnesses and their families by addressing their physical, psychological, social, and spiritual needs. Effective communication, symptom management, addressing psychological and spiritual needs, respecting patients' autonomy, providing patient and family education, cultural and linguistic competence, and healthcare provider competence are all crucial aspects of palliative care that can improve patient's quality of life and reduce their suffering. We also highlight the practicality of the Palliative Outreach program as a model for integrating palliative care into general services to serve oncology and non-oncology terminal patients. Finally, palliative care should be offered based on need rather than diagnosis, setting, economic restrictions, or cultural perspective. Future research is needed to understand further the unique needs and challenges of palliative care in different cultural and societal contexts.

## References

[1] Graham F, Clark D (2007). The changing model of palliative care. Medicine.

[2] Claessen SJ, Francke AL, Sixma HJ, de Veer AJ, Deliens L (2012). Measuring patients' experiences with palliative care: the Consumer Quality Index Palliative Care. BMJ Support Palliat Care.

[3] van Campen C, Sixma H, Friele RD, Kerssens JJ, Peters L (1995). Quality of care and patient satisfaction: a review of measuring instruments. Med Care Res Rev.

[4] Sixma HJ, Kerssens JJ, Campen CV, Peters L (1998). Quality of care from the patients' perspective: from theoretical concept to a new measuring instrument. Health Expect.

[5] Hopman P, de Boer D, Rademakers J (2011). Kennisvraag: wat heeft vijf jaar CQ-index opgeleverd? [Dutch]. NIVEL.

[6] Luckett T, Phillips J, Agar M, Virdun C, Green A, Davidson PM (2014). Elements of effective palliative care models: a rapid review. BMC Health Serv Res.

[7] Zalenski RJ, Jones SS, Courage C (2017). Impact of palliative care screening and consultation in the ICU: a multihospital quality improvement project. J Pain Symptom Manage.

[8] Murphy E, Froggatt K, Connolly S, O'Shea E, Sampson EL, Casey D, Devane D (2016). Palliative care interventions in advanced dementia. Cochrane Database Syst Rev.

[9] Potts M, Cartmell KB, Nemeth LS, Qanungo S (2019). A qualitative evaluation of a home-based palliative care program utilizing community health workers in India. Indian J Palliat Care.

[10] Fulton JJ, LeBlanc TW, Cutson TM (2019). Integrated outpatient palliative care for patients with advanced cancer: a systematic review and meta-analysis. Palliat Med.

[11] (2023). Palliative care. https://www.who.int/health-topics/palliative-care.

[12] Ferrell BR, Temel JS, Temin S (2017). Integration of palliative care into standard oncology care: American Society of Clinical Oncology clinical practice guideline update. J Clin Oncol.

[13] McIlvennan CK, Allen LA (2016). Palliative care in patients with heart failure. BMJ.

[14] Schenker Y, Arnold R (2015). The next era of palliative care. JAMA.

[15] Back A, Tulsky JA, Arnold RM (2020). Communication skills in the age of COVID-19. Ann Intern Med.

[16] Cherny NI, Portenoy RK (1994). The management of cancer pain. CA Cancer J Clin.

[17] (2023). National Comprehensive Cancer Network. NCCN Clinical Practice Guidelines in Oncology (NCCN Guidelines®). Adult Cancer Pain, Version 3. https://www.nccn.org/professionals/physician_gls/pdf/pain.pdf.

[18] Breitbart W (2002). Spirituality and meaning in supportive care: spirituality- and meaning-centered group psychotherapy interventions in advanced cancer. Support Care Cancer.

[19] Sorato DB, Osório FL (2015). Coping, psychopathology, and quality of life in cancer patients under palliative care. Palliat Support Care.

[20] Puchalski CM, Vitillo R, Hull SK, Reller N (2014). Improving the spiritual dimension of whole person care: reaching national and international consensus. J Palliat Med.

[21] Balboni TA, Vanderwerker LC, Block SD, Paulk ME, Lathan CS, Peteet JR, Prigerson HG (2007). Religiousness and spiritual support among advanced cancer patients and associations with end-of-life treatment preferences and quality of life. J Clin Oncol.

[22] Nandan M S, Montshiwa C, Sneha N S Rene, A A (2017). Spiritual pain: should medical professionals do spiritual care? if yes, how?. Canc Therapy & Oncol Int J.

[23] Houska A, Loučka M (2019). Patients' autonomy at the end of life: a critical review. J Pain Symptom Manage.

[24] Kuosmanen L, Hupli M, Ahtiluoto S, Haavisto E (2021). Patient participation in shared decision-making in palliative care - an integrative review. J Clin Nurs.

[25] (2023). National Hospice and Palliative Care Organization. Hospice and Palliative Care: Patient and Family Education. https://www.nhpco.org/wp-content/uploads/2019/05/Patient-and-Family-Education.pdf.

[26] Betancourt JR, Green AR, Carrillo JE (2002). Cultural competence in health care: emerging frameworks and practical approaches. field report.

[27] Brach C, Fraser I (2000). Can cultural competency reduce racial and ethnic health disparities? a review and conceptual model. Med Care Res Rev.

[28] Berwick DM, Nolan TW, Whittington J (2008). The triple aim: care, health, and cost. Health Aff (Millwood).

